# Admission Blood Biomarkers for Differentiating Acute Appendicitis from Acute Enteritis in Children Younger than Five Years: A Retrospective Cohort Study

**DOI:** 10.3390/medsci14060673

**Published:** 2026-10-14

**Authors:** Jana Buzuk, Bianka Dujić, Sara Vuković, Marko Bašković

**Affiliations:** 1 School of Medicine, Catholic University of Croatia, Ilica 242, 10000 Zagreb, Croatia; 2 Department of Pediatric Surgery, Children’s Hospital Zagreb, Ulica Vjekoslava Klaića 16, 10000 Zagreb, Croatia; 3 School of Medicine, University of Zagreb, Šalata 3, 10000 Zagreb, Croatia; 4 Scientific Centre of Excellence for Reproductive and Regenerative Medicine, School of Medicine, University of Zagreb, Šalata 3, 10000 Zagreb, Croatia; 5 Croatian Academy of Medical Sciences, Kaptol 15, 10000 Zagreb, Croatia

**Keywords:** acute appendicitis, acute enteritis, children, blood biomarkers, neutrophil-to-lymphocyte ratio, C-reactive protein, differential diagnosis

## Abstract

**Background/Objectives:** Acute abdominal pain in children younger than five years is a diagnostic challenge because acute appendicitis and acute enteritis share overlapping clinical features, and delayed recognition of appendicitis increases the risk of perforation and postoperative complications. We aimed to identify admission blood biomarkers that reliably distinguish these two conditions and to quantify their diagnostic accuracy. **Methods:** We conducted a retrospective observational cohort study at the Children’s Hospital Zagreb covering the period from 1 January 2016 to 1 January 2026. Children younger than five years with a confirmed diagnosis of acute appendicitis (Group A, *n* = 80) or acute enteritis (Group B, *n* = 66) were included. Appendicitis was confirmed intraoperatively and histopathologically, whereas enteritis was confirmed by stool testing, imaging, and clinical course. The complete blood count with differential, derived inflammatory indices, and C-reactive protein obtained on admission were compared using the Mann–Whitney U test, receiver operating characteristic analysis, and multivariable logistic regression. **Results:** Appendicitis was associated with higher neutrophil percentage, neutrophil-to-lymphocyte ratio, C-reactive protein, and composite systemic inflammatory indices, whereas enteritis showed higher lymphocyte percentage, eosinophil percentage, and platelet count. The strongest single discriminators were C-reactive protein (area under the curve 0.91), neutrophil percentage (0.90), and the neutrophil-to-lymphocyte ratio (0.90). A multivariable model combining log-transformed C-reactive protein and neutrophil-to-lymphocyte ratio reached an apparent area under the curve of 0.96, with an optimism-corrected value of 0.95, and 96.3% sensitivity and 86.4% specificity, and both markers were independent predictors of appendicitis. Children with appendicitis also more often showed rebound tenderness and right lower quadrant tenderness, complicated appendicitis was present in 78.8% of them, and among operated children, a higher immature granulocyte fraction and count were associated with complicated disease in an exploratory analysis. **Conclusions:** Simple and inexpensive blood biomarkers available on admission, in particular C-reactive protein and the neutrophil-to-lymphocyte ratio, are strongly associated with the final diagnosis and discriminate acute appendicitis from acute enteritis in children younger than five years. Prospective validation in consecutive children with diagnostic uncertainty is needed before they can be used to guide clinical decisions.

## 1. Introduction

Acute appendicitis is the most common abdominal surgical emergency in childhood [1]. In children younger than five years, however, it remains one of the most difficult diagnoses in everyday pediatric practice. Young children cannot localize or describe their pain; the clinical picture is dominated by nonspecific symptoms such as vomiting, diarrhea, and fever, and the physical examination is often unreliable. For these reasons, appendicitis in preschool children is frequently recognized late and carries high rates of perforation, abscess formation, and prolonged hospitalization [2,3,4].

Acute enteritis, by contrast, is far more common in this age group and is one of the leading causes of morbidity and hospital admission in children younger than five years worldwide [5]. Because appendicitis in young children often begins with diarrhea and vomiting, it can closely mimic enteritis, and the two conditions are easily confused at first contact [6]. Misclassifying early appendicitis as enteritis delays surgery and increases the risk of complications, whereas overcalling appendicitis in a child who actually has enteritis leads to unnecessary imaging, admission, and operative exploration [6,7].

Clinical scoring systems such as the Alvarado score and the Pediatric Appendicitis Score were developed to structure this decision, but they were derived mainly from older children and perform poorly in the preschool group [8,9,10]. Imaging is useful yet limited, because ultrasound is operator-dependent and the appendix is frequently not visualized in small children, while computed tomography raises concerns about ionizing radiation [11]. There is therefore strong interest in simple laboratory markers that are available at the moment of admission and can help separate a surgical from a nonsurgical cause of acute abdominal pain.

The complete blood count with differential and C-reactive protein are obtained routinely in almost every child who presents with acute abdominal pain. Beyond the individual parameters, ratios and composite indices derived from the same count, such as the neutrophil-to-lymphocyte ratio, the platelet-to-lymphocyte ratio, the monocyte-to-lymphocyte ratio, the systemic immune-inflammation index, and the systemic inflammatory response index, have emerged as inexpensive measures of the systemic inflammatory response and have shown promise in the diagnosis of appendicitis [12,13,14,15]. Most of these studies, however, compared appendicitis with healthy controls or with children who had nonspecific abdominal pain, and very few addressed the specific and clinically relevant question of separating appendicitis from enteritis in the youngest patients.

The aim of this study was to identify admission blood biomarkers that differentiate confirmed acute appendicitis from confirmed acute enteritis in children younger than five years. Our primary objective was to identify the laboratory biomarkers measured on the day of admission that best discriminate between the two conditions. Our secondary objective was to quantify their diagnostic performance in terms of sensitivity, specificity, and predictive values and to identify independent predictors through multivariable analysis.

## 2. Materials and Methods

### 2.1. Study Design and Setting

**Figure 1:**
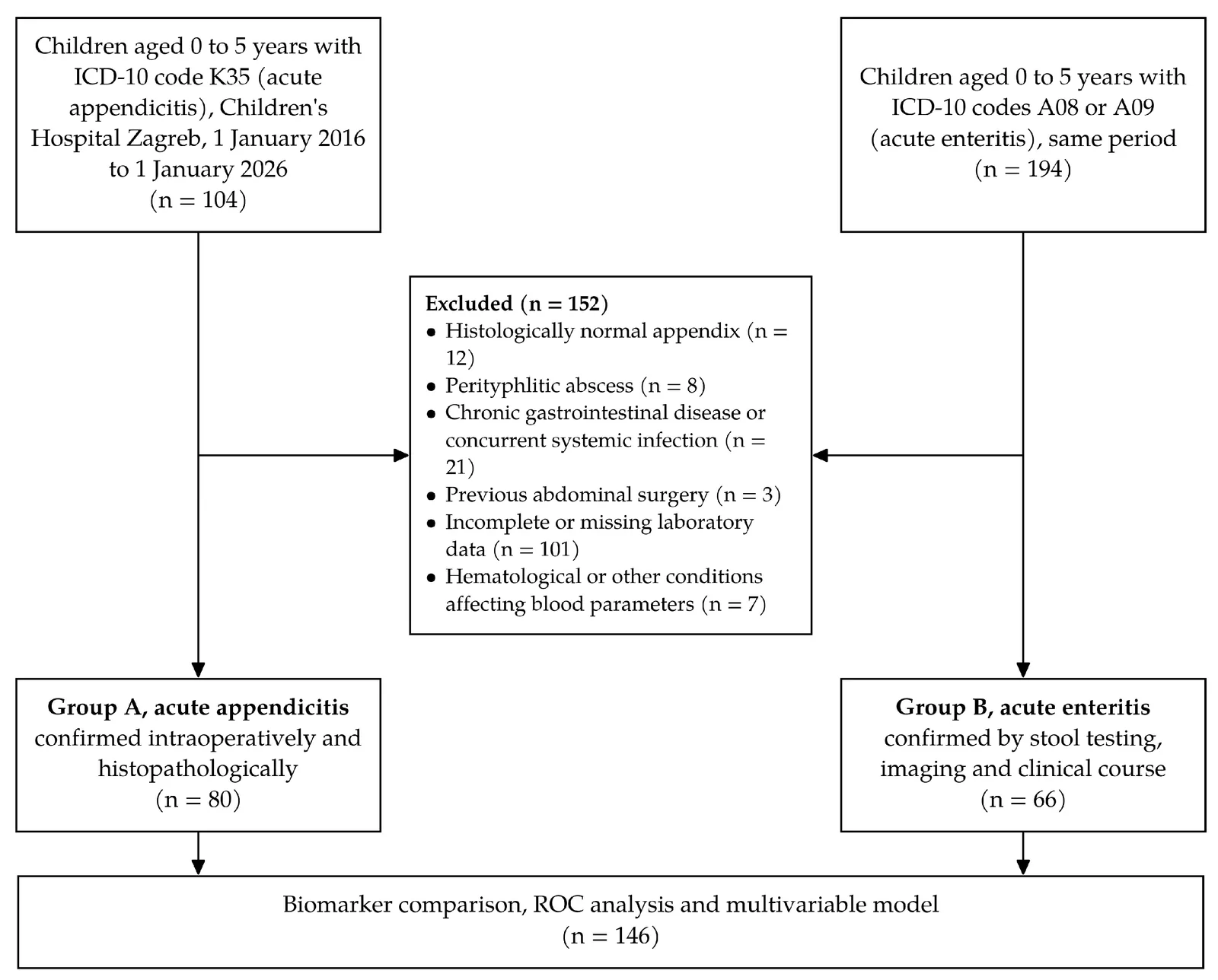
Flow of participants through the study.

This was a retrospective observational cohort study conducted at the Children’s Hospital Zagreb, a tertiary pediatric center. Electronic patient records held in the hospital information system (IN2 BIS^®^, *version 216.0.000*, Constellation Software Inc., Toronto, ON, Canada) were reviewed for the ten-year period from 1 January 2016 to 1 January 2026. Throughout the study period, eligible cases were identified from these records by their International Classification of Diseases, tenth revision code, namely K35 with all subcodes for acute appendicitis and A08 and A09 for acute enteritis, and for each child a predefined set of demographic, clinical, and laboratory variables was extracted into a standardized dataset using a single consistent protocol for case ascertainment, data extraction, and analysis. Reporting adheres to the Strengthening the Reporting of Observational Studies in Epidemiology guidance for observational research [16]. All consecutive children who met the eligibility criteria during the study period were included, and no matching or additional sampling was performed. The two diagnostic groups were identified independently according to the final diagnosis rather than from a single cohort of children investigated for suspected appendicitis. The flow of participants through the study is shown in Figure 1.

### 2.2. Participants and Group Definition

We included children younger than five years who were admitted with acute abdominal or gastrointestinal symptoms and in whom a final diagnosis of acute appendicitis or acute enteritis was confirmed. Children were assigned to one of two groups. Group A comprised children with confirmed acute appendicitis, and Group B comprised children with confirmed acute enteritis. Appendicitis was established intraoperatively by the pediatric surgeon and confirmed by histopathological examination of the resected appendix. Appendicitis was further classified as complicated when the histopathological findings showed gangrenous appendicitis, perforated gangrenous appendicitis, empyema, or a periappendicular infiltrate, and as uncomplicated otherwise. Enteritis was based on stool testing, imaging when performed, and a compatible clinical course that resolved under conservative management. The original histopathology reports issued by different pathologists in routine practice were used without central re-evaluation, and the histopathological report was taken as the reference standard for the classification of disease. Children who underwent appendectomy with a histologically normal appendix were excluded. Nonoperative antibiotic treatment of appendicitis is not used at our institution, so all children with appendicitis were treated surgically. The causative agent of enteritis was sought in stool samples. In children without an identified pathogen, enteritis was diagnosed clinically on the basis of a typical presentation with diarrhea and vomiting and the resolution of symptoms under supportive treatment, and none of these children was subsequently operated on or readmitted to our institution with appendicitis.

Inclusion criteria were an age younger than five years, admission with acute abdominal or gastrointestinal symptoms, a confirmed diagnosis of acute appendicitis or acute enteritis on the basis of clinical, imaging, surgical, and histopathological data, and blood sampling performed on the day of admission before in-hospital treatment. Exclusion criteria were a known chronic gastrointestinal disease or another concurrent systemic infection, previous abdominal surgery, incomplete or missing laboratory data, coexisting conditions that affect blood parameters such as hematological disorders, and a perityphlitic abscess. Children with a perityphlitic abscess were excluded because it represents a distinct, later stage of the disease that is often managed differently from acute appendicitis.

At presentation, children were first examined by a resident or specialist in pediatrics or pediatric surgery, and the final decision to operate was always made by a specialist in pediatric surgery. Abdominal ultrasound performed by a radiologist was the first-line imaging modality in suspected appendicitis, whereas in children with enteritis it was performed only when the clinical picture was unclear. Computed tomography is generally not used in this age group at our institution, and point-of-care ultrasound was not used. Urinalysis was obtained routinely, and no child included in the study had a urinary tract infection.

### 2.3. Data Collection and Laboratory Analysis

For each child, we recorded sex, age in months, the month and time of admission, the duration of symptoms, and the clinical findings at presentation, together with the mode of management and its outcome. Blood was drawn on the day of admission, before any in-hospital treatment, at presentation to the emergency department, obtained by a nurse or a laboratory technician following standard procedures. The interval between symptom onset and blood sampling therefore corresponds to the reported duration of symptoms. The complete blood count with differential was analyzed on an XN-1000 automated hematology analyzer (Sysmex Corporation, Kobe, Japan). C-reactive protein was measured on a D7-CRP hematology analyzer (Dymind Biotechnology, Shenzhen, China) or on an AU400 or AU680 clinical chemistry analyzer (Beckman Coulter, Brea, CA, USA).

The parameters obtained on admission were erythrocytes, hemoglobin, hematocrit, mean corpuscular volume, mean corpuscular hemoglobin, mean corpuscular hemoglobin concentration, red cell distribution width, platelets, mean platelet volume, C-reactive protein, leukocytes, and the neutrophil, lymphocyte, monocyte, eosinophil, basophil, and immature granulocyte fractions expressed both as percentages and as absolute counts. From these values we calculated the neutrophil-to-lymphocyte ratio, the platelet-to-lymphocyte ratio, the monocyte-to-lymphocyte ratio, the neutrophil-to-monocyte ratio, the systemic immune-inflammation index defined as platelets multiplied by neutrophils divided by lymphocytes, and the systemic inflammatory response index defined as neutrophils multiplied by monocytes divided by lymphocytes.

Intraoperative peritoneal fluid swabs were sent for microbiological culture. When perioperative antibiotic treatment was indicated, usually cefuroxime combined with metronidazole, it was chosen by the operating surgeon, and its duration was decided by the attending physician according to the operative findings and the clinical course. Omentectomy was performed at the discretion of the surgeon when the omentum was necrotic or firmly adherent to the inflamed appendix as part of a periappendicular inflammatory mass. After surgery for complicated, and in particular perforated, appendicitis, children were admitted to the intensive care unit at the discretion of the surgeon for close monitoring because young children have a limited physiological reserve and may deteriorate rapidly into sepsis and because they often require intensive fluid resuscitation and analgesia in the early postoperative period.

### 2.4. Statistical Analysis

Continuous variables are summarized as the median with the interquartile range, and categorical variables as counts with percentages. Because most hematological indices departed from a normal distribution, continuous variables were compared between the two groups with the Mann–Whitney U test, and categorical variables with the chi-square test or the Fisher exact test as appropriate. All laboratory parameters were available for every child, so all biomarker analyses and the multivariable model included the complete study sample. To account for the large number of biomarkers examined, the *p* values from the biomarker comparisons were corrected for the false discovery rate with the Benjamini–Hochberg procedure, and the corrected value is reported as q. The diagnostic performance of each biomarker was assessed by receiver operating characteristic analysis, with appendicitis coded as the positive outcome. The area under the curve is reported together with the optimal cutoff selected by the Youden index and with the sensitivity, specificity, and positive and negative predictive values at that cutoff. A test was considered positive when the value was at or above the cutoff for markers that were higher in appendicitis and at or below the cutoff for markers that were lower in appendicitis. Predictive values were calculated at the observed prevalence of appendicitis in the study sample and therefore cannot be transferred directly to populations with a different prevalence. The 95% confidence intervals of the area under the curve were calculated with the Hanley and McNeil method, and those of sensitivity, specificity, and predictive values with the Wilson method. Independent predictors of appendicitis were identified by multivariable binary logistic regression, with results expressed as adjusted odds ratios per one standard deviation increase and their 95% confidence intervals, and the number of predictors was deliberately kept small to respect the events per variable principle. The predictors were selected on the basis of their discriminative performance and clinical availability, with C-reactive protein representing the acute phase response and the neutrophil-to-lymphocyte ratio as the strongest index derived from the differential count. Both were entered as continuous variables. Because the Box-Tidwell procedure indicated departure from linearity in the logit for the untransformed values, both predictors were natural log-transformed, and linearity was then rechecked with the same procedure. The odds ratios per one standard deviation were obtained by multiplying each coefficient by the standard deviation of the log-transformed predictor in the model sample, and collinearity was assessed with the correlation coefficient and the variance inflation factor. The probability threshold for classification was chosen with the Youden index. Internal validation was performed with 500 bootstrap resamples, in which the model was refitted in each resample, to estimate the optimism in the area under the curve and the calibration slope, and calibration was further assessed with the Hosmer–Lemeshow test and the Brier score. In sensitivity analyses, the model was refitted with untransformed predictors, symptom duration and prehospital antibiotic use were added to the model, and the incremental value of the two biomarkers over a clinical model containing abdominal pain, right lower quadrant tenderness, and diarrhea was assessed with the likelihood ratio test. The model was not adjusted for age because the two groups were of similar age. Admission biomarkers were also compared between complicated and uncomplicated appendicitis with the same nonparametric and receiver operating characteristic methods, uncomplicated appendicitis was compared with enteritis in an exploratory analysis, and the clinical and historical variables were compared between the two diagnostic groups. Analyses were performed in XLSTAT^®^ (version 2020.5.1, Addinsoft, Paris, France) within Microsoft Excel^®^ for Windows (Microsoft Corporation, Redmond, WA, USA), and a two-sided *p*-value below 0.05 was considered statistically significant.

## 3. Results

### 3.1. Baseline Characteristics of the Study Population

A total of 146 children younger than five years met the inclusion criteria, 80 with confirmed acute appendicitis (Group A) and 66 with confirmed acute enteritis (Group B). Boys accounted for 51 of 80 children (63.8%) in Group A and for 37 of 66 children (56.1%) in Group B. Children with appendicitis and children with enteritis were of similar age, with a median age of 49 months (interquartile range 40 to 54) compared with 47 months (interquartile range 38 to 53). Body temperature on admission did not differ significantly between the groups. The baseline characteristics are shown in Table 1.

### 3.2. Clinical Presentation and History

The two groups differed clearly in their presenting features. Abdominal pain, right lower quadrant tenderness, and rebound tenderness were far more common in children with appendicitis, and rebound tenderness was recorded only in the appendicitis group. Diarrhea and bloody stools were more frequent in children with enteritis, whereas nausea and vomiting, poor appetite, and decreased urination did not differ between the groups. The clinical and historical features are shown in Table 2.

### 3.3. Seasonal and Temporal Distribution

Admissions occurred throughout the year in both groups. Appendicitis was distributed fairly evenly across the four seasons, whereas enteritis was somewhat more frequent in spring, but the overall seasonal distribution did not differ significantly between the groups. In contrast, children with appendicitis were admitted during daytime hours more often than children with enteritis (62.5% versus 39.4%, *p* = 0.008). The seasonal and temporal pattern of admissions is summarized in Table 3.

### 3.4. Management and In-Hospital Outcomes

Management and in-hospital outcomes reflected the surgical nature of appendicitis. All 80 children with appendicitis underwent appendectomy, and the approach was open in 77 children (96.3%) and laparoscopic in 3 children (3.8%). Complicated appendicitis was present in 63 children (78.8%). Histopathology showed gangrenous appendicitis without perforation in 24 children and perforated gangrenous appendicitis in 38 children, including a covered perforation in 2. Periappendicular empyema was present in 6 children, in 5 of them together with gangrenous appendicitis without perforation, and a periappendicular infiltrate was present in 1 child with gangrenous appendicitis. The remaining 17 children had uncomplicated, mostly phlegmonous, appendicitis. The proportion of complicated disease therefore reflects the full histopathological spectrum of complicated disease and should not be read as a perforation rate. The appendix was retrocecal in 4 children and subhepatic in 1 child, and none of these five children had decreased urination. Abdominal drainage was required in 38 children (47.5%), omentectomy in 16 children (20.0%), and admission to the intensive care unit in 40 children (50.0%). Antibiotic therapy was given to 63 operated children (78.8%) in the perioperative and postoperative period. Children with enteritis were managed conservatively, and only 13 of them (19.7%) received antibiotics. The length of hospital stay was markedly longer in the appendicitis group, with a median of 9 days (IQR 6–11.5) compared with 3 days (IQR 2–4.75) in the enteritis group (*p* < 0.001). Prehospital antibiotic treatment had been received by 32 of 80 children (40.0%) with appendicitis and by 6 of 66 children (9.1%) with enteritis. No child in either group died.

Stool testing identified a causative pathogen in 49 of 66 children (74.2%) with enteritis. Rotavirus was by far the most common agent (26 children), followed by astrovirus (8 children), norovirus (6 children), and adenovirus (2 children), with sapovirus in 1 child. Bacterial pathogens were found in 9 children, and parasites in 2 children. Mixed infections were present in some children, and no pathogen was identified in 17 children.

### 3.5. Comparison of Admission Blood Biomarkers

Acute appendicitis was associated with a pronounced neutrophilic response. The neutrophil percentage, the absolute neutrophil count, the leukocyte count, the neutrophil-to-lymphocyte ratio, the neutrophil-to-monocyte ratio, the monocyte-to-lymphocyte ratio, the platelet-to-lymphocyte ratio, the systemic immune-inflammation index, the systemic inflammatory response index, the immature granulocyte fraction and absolute count, and C-reactive protein were all significantly higher in Group A than in Group B. In contrast, the lymphocyte percentage and absolute count, the eosinophil percentage and absolute count, the monocyte percentage, the basophil percentage, and the platelet count were significantly higher in Group B. C-reactive protein showed the widest separation, with a median of 81.2 mg/L (interquartile range 56.6 to 111.9) in appendicitis compared with 4.0 mg/L (interquartile range 0.5 to 13.5) in enteritis.

The erythrocyte count was slightly higher in the appendicitis group. The red cell indices, namely the mean corpuscular volume, the mean corpuscular hemoglobin, and the red cell distribution width, were higher in the enteritis group. Hemoglobin, hematocrit, mean corpuscular hemoglobin concentration, mean platelet volume, and the absolute monocyte and basophil counts did not differ significantly between the groups. All comparisons, with the medians, interquartile ranges, and false discovery rate-corrected q values, are presented in Table 4.

### 3.6. Diagnostic Performance of Individual Biomarkers

**Figure 2:**
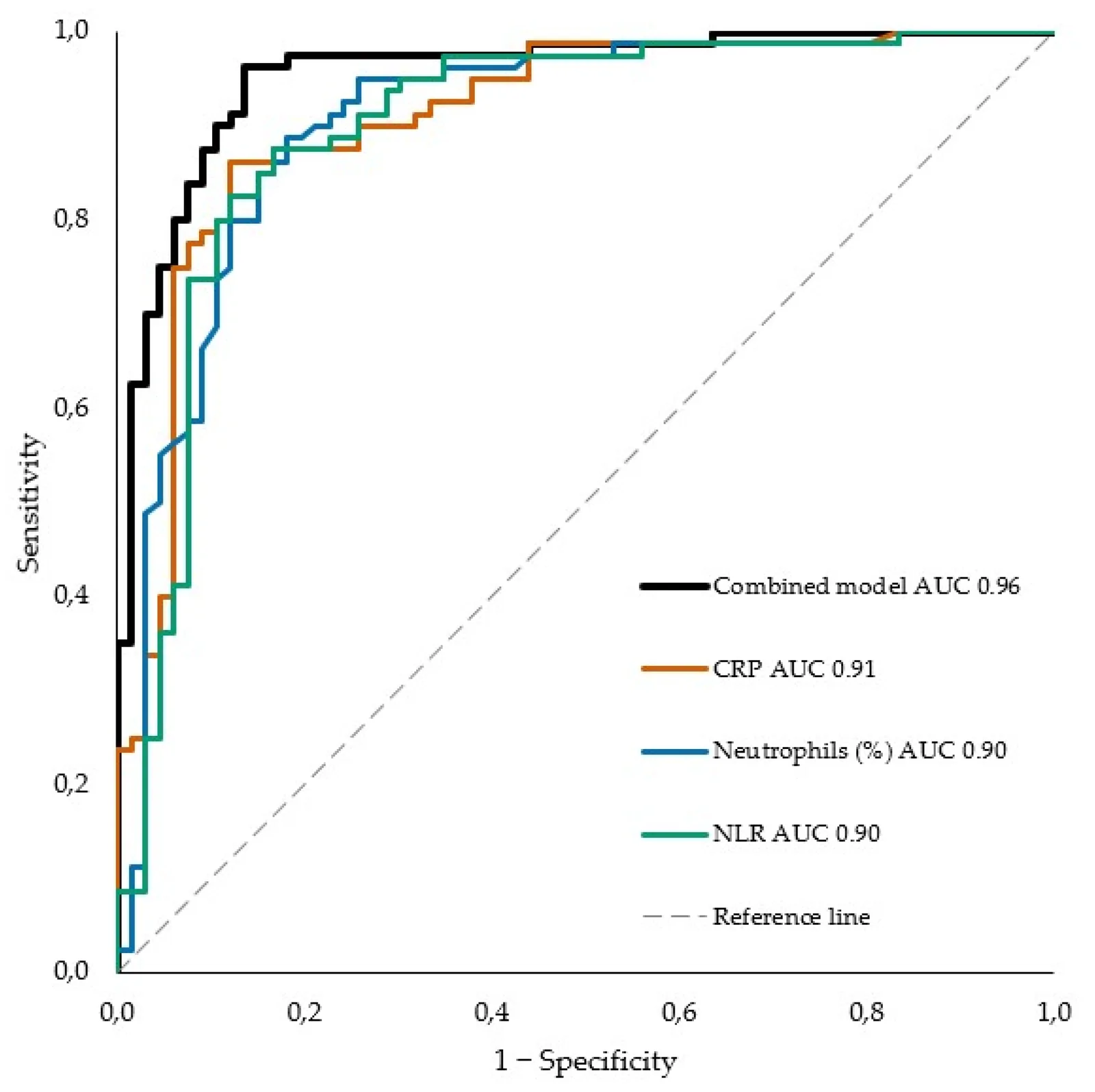
Receiver operating characteristic curves of the leading admission biomarkers and of the combined model for differentiating acute appendicitis from acute enteritis. The areas under the curve with 95% confidence intervals were 0.96 (0.92 to 0.99) for the combined model of log-transformed C-reactive protein and neutrophil-to-lymphocyte ratio, 0.91 (0.86 to 0.96) for C-reactive protein (CRP), 0.90 (0.85 to 0.95) for the neutrophil percentage, and 0.90 (0.85 to 0.95) for the neutrophil-to-lymphocyte ratio (NLR). The dashed diagonal line represents no discrimination.

**Figure 3:**
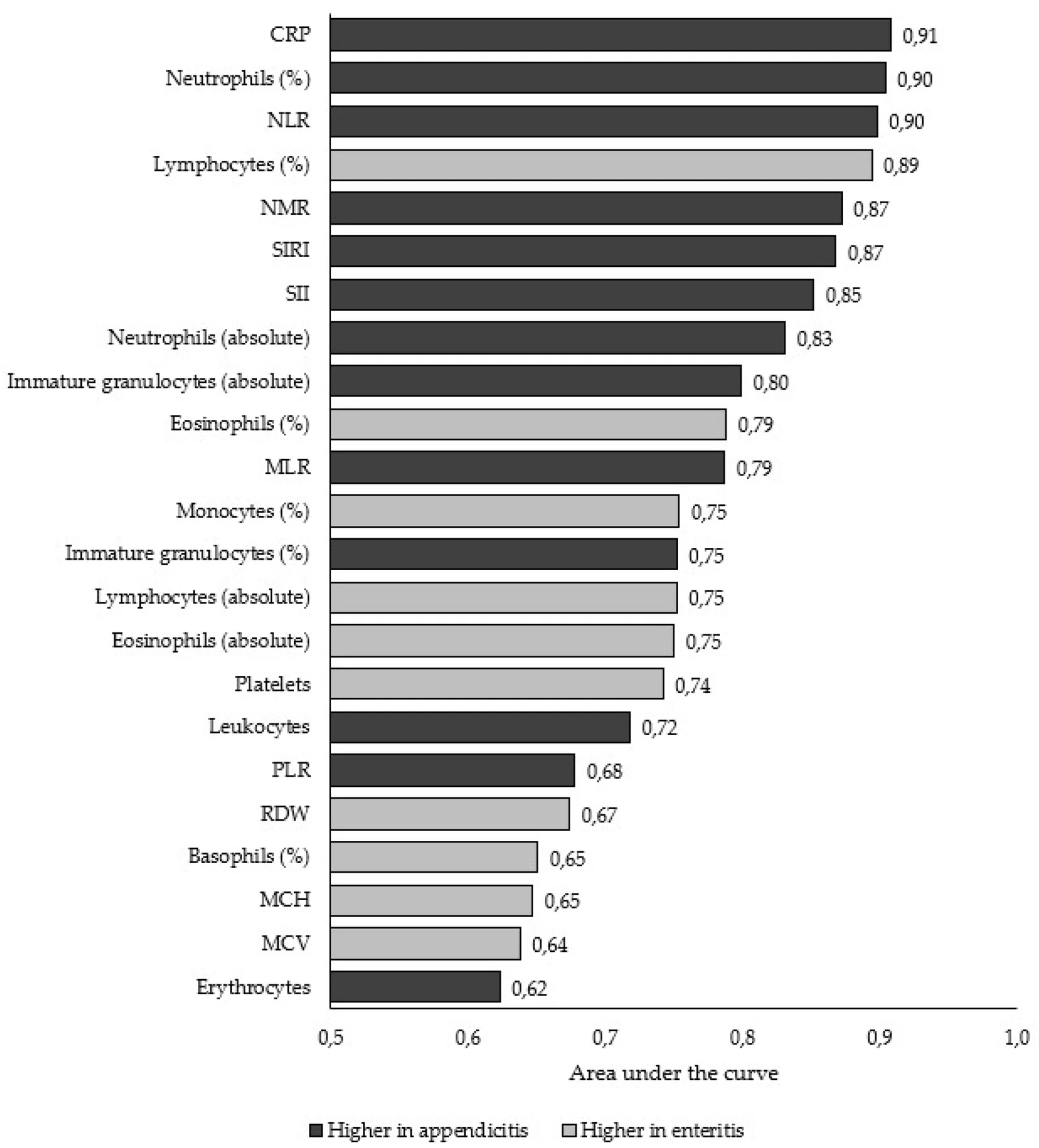
Area under the receiver operating characteristic curve for the 23 admission biomarkers that significantly differentiated acute appendicitis from acute enteritis after false discovery rate correction (q < 0.05), ranked in descending order. Dark bars indicate markers higher in appendicitis, and light bars indicate markers higher in enteritis, for which the area under the curve is expressed for lower values predicting appendicitis. CRP, C-reactive protein. NLR, neutrophil-to-lymphocyte ratio. NMR, neutrophil-to-monocyte ratio. SIRI, systemic inflammatory response index. SII, systemic immune-inflammation index. MLR, monocyte-to-lymphocyte ratio. PLR, platelet-to-lymphocyte ratio. RDW, red cell distribution width. MCH, mean corpuscular hemoglobin. MCV, mean corpuscular volume.

On receiver operating characteristic analysis, the strongest single discriminators of appendicitis were C-reactive protein with an area under the curve of 0.91, the neutrophil percentage and the neutrophil-to-lymphocyte ratio, each at 0.90, followed by the lymphocyte percentage at 0.89, the neutrophil-to-monocyte ratio and the systemic inflammatory response index, each at 0.87, and the systemic immune-inflammation index at 0.85. At the optimal cutoff, a neutrophil percentage of 65% or higher identified appendicitis with 88.8% sensitivity and 81.8% specificity, a neutrophil-to-lymphocyte ratio of 3.12 or higher with 87.5% sensitivity and 83.3% specificity, and C-reactive protein of 25.3 mg/L or higher with 86.3% sensitivity and 87.9% specificity. The areas under the curve were 0.90 (95% confidence interval 0.85 to 0.95) for the neutrophil percentage, 0.90 (0.85 to 0.95) for the neutrophil-to-lymphocyte ratio, and 0.91 (0.86 to 0.96) for C-reactive protein. At these cutoffs the neutrophil percentage correctly classified 71 of 80 children with appendicitis and 54 of 66 children with enteritis, the neutrophil-to-lymphocyte ratio 70 of 80 and 55 of 66, and C-reactive protein 69 of 80 and 58 of 66, respectively. The corresponding 95% confidence intervals were 80.0 to 94.0 for the sensitivity and 70.9 to 89.3 for the specificity of the neutrophil percentage, 78.5 to 93.1 and 72.6 to 90.4 for the neutrophil-to-lymphocyte ratio, and 77.0 to 92.1 and 77.9 to 93.7 for C-reactive protein. The positive and negative predictive values were 85.5% (76.4 to 91.5) and 85.7% (75.0 to 92.3) for the neutrophil percentage, 86.4% (77.3 to 92.2) and 84.6% (73.9 to 91.4) for the neutrophil-to-lymphocyte ratio, and 89.6% (80.8 to 94.6) and 84.1% (73.7 to 90.9) for C-reactive protein. The lymphocyte percentage discriminated almost as well in the opposite direction, being lower in appendicitis. The diagnostic metrics of the biomarkers with an area under the curve of at least 0.70 are summarized in Table 5, and the receiver operating characteristic curves of the leading markers and of the combined model are shown in Figure 2, and the areas under the curve for all significant biomarkers are ranked in descending order in Figure 3.

Cutoffs are expressed in the units of each biomarker, namely % for the percentage fractions, ×10^9^/L for absolute cell counts and platelets, mg/L for C-reactive protein, and dimensionless values for the ratios and composite indices. A test was considered positive at or above the cutoff (≥) for markers higher in appendicitis and at or below the cutoff (≤) for markers higher in enteritis. Appendicitis was coded as the positive outcome, and predictive values refer to the observed prevalence in the study sample. AUC, area under the curve. PPV, positive predictive value. NPV, negative predictive value.

### 3.7. Independent Predictors and the Combined Model

In the multivariable logistic regression model, both C-reactive protein and the neutrophil-to-lymphocyte ratio were independent predictors of appendicitis, with adjusted odds ratios per one standard deviation increase in the log-transformed value of 7.77 (95% confidence interval 3.25 to 18.55) and 8.55 (95% confidence interval 3.43 to 21.31), respectively. The fitted equation was logit(p) = −4.7608 + 0.9000 × ln[C-reactive protein (mg/L)] + 1.9451 × ln[neutrophil-to-lymphocyte ratio], where ln is the natural logarithm and p is the predicted probability of appendicitis. The means and standard deviations of the log-transformed predictors used for scaling the odds ratios were 2.727 and 2.278 for C-reactive protein and 1.110 and 1.103 for the neutrophil-to-lymphocyte ratio. At a predicted probability threshold of 0.422, the model correctly classified 77 of 80 children with appendicitis and 57 of 66 children with enteritis. The combined model separated appendicitis from enteritis with an apparent area under the curve of 0.96 (95% confidence interval 0.92 to 0.99), a sensitivity of 96.3% (89.5 to 98.7), a specificity of 86.4% (76.1 to 92.7), a positive predictive value of 89.5% (81.3 to 94.4), and a negative predictive value of 95.0% (86.3 to 98.3). After bootstrap internal validation, the optimism-corrected area under the curve was 0.95, and the calibration slope was 0.91. The Hosmer–Lemeshow test showed no significant lack of fit (*p* = 0.23), and the Brier score was 0.076. There was no relevant collinearity between the two log-transformed predictors (r = 0.52, variance inflation factor 1.37). The Box-Tidwell test indicated departure from linearity for the untransformed predictors (*p* = 0.004 for C-reactive protein and *p* = 0.002 for the neutrophil-to-lymphocyte ratio) but not after log transformation (*p* = 0.32 and *p* = 0.36, respectively). A model with untransformed predictors gave a similar apparent area under the curve of 0.95 (0.91 to 0.98). Adding symptom duration did not change the model (*p* = 0.43). Prehospital antibiotic use was itself associated with appendicitis, but both C-reactive protein and the neutrophil-to-lymphocyte ratio remained independent predictors after its inclusion. Compared with a clinical model containing abdominal pain, right lower quadrant tenderness, and diarrhea, which reached an area under the curve of 0.94, the addition of C-reactive protein and the neutrophil-to-lymphocyte ratio increased the area under the curve to 0.99 (likelihood ratio test *p* < 0.001). Adding the platelet count as a third predictor did not materially improve the model, and replacing the neutrophil-to-lymphocyte ratio with the neutrophil percentage yielded a comparable area under the curve of 0.96. The adjusted odds ratios are presented in Table 6, and the intercept and regression coefficients are given in its footnote.

### 3.8. Admission Biomarkers and Complicated Appendicitis

Within the appendicitis group we examined whether admission biomarkers could separate complicated from uncomplicated disease. Most inflammatory markers, including C-reactive protein, the neutrophil-to-lymphocyte ratio, the neutrophil percentage, and the composite indices, did not differ significantly between complicated and uncomplicated appendicitis. The immature granulocyte fraction and absolute count were the exception and were higher in complicated disease (*p* = 0.008 and *p* = 0.026, respectively). The absolute immature granulocyte count reached an area under the curve of 0.68 (95% confidence interval 0.54 to 0.81) at a cutoff of 0.06 × 10^9^/L or higher, with a sensitivity of 68.3% (43 of 63, 95% confidence interval 56.0 to 78.4) and a specificity of 64.7% (11 of 17, 95% confidence interval 41.3 to 82.7). After correction for multiple testing, neither difference remained significant (q = 0.15 for the fraction and q = 0.25 for the absolute count), so this finding should be regarded as exploratory. Children with complicated appendicitis also remained in hospital longer. These findings are shown in Table 7.

In an exploratory comparison restricted to the 17 children with uncomplicated appendicitis and the 66 children with enteritis, the neutrophil percentage and the neutrophil-to-lymphocyte ratio still separated uncomplicated appendicitis from enteritis, with areas under the curve of 0.93 and 0.92, respectively, while C-reactive protein reached 0.87.

## 4. Discussion

In this retrospective cohort of children younger than five years, admission blood biomarkers clearly separated acute appendicitis from acute enteritis. Appendicitis was characterized by a neutrophil-dominant inflammatory response, with a high neutrophil percentage, a high neutrophil-to-lymphocyte ratio, elevated composite inflammatory indices, and markedly raised C-reactive protein, whereas enteritis was characterized by relative lymphocytosis, higher eosinophil counts, and higher platelet counts. The neutrophil percentage, the neutrophil-to-lymphocyte ratio, and C-reactive protein were the strongest individual discriminators, and a simple model that combined C-reactive protein with the neutrophil-to-lymphocyte ratio achieved high apparent discrimination with an area under the curve of 0.96 in the development sample, which remained 0.95 after internal validation.

The clinical features recorded on admission reinforced the laboratory findings. Rebound tenderness was present only in children with appendicitis, and right lower quadrant tenderness was almost twice as common in that group, whereas diarrhea and bloody stools were far more frequent in enteritis. These observations agree with long-standing reports that localized peritoneal signs are difficult to elicit in very young children and that diarrhea is a common and misleading feature of appendicitis in this age group [17,18]. Because vomiting, poor appetite, and fever overlapped substantially between the two conditions, no single symptom reliably separated them, which underlines the potential role of objective admission biomarkers in this setting [19].

The seasonal distribution of admissions did not differ significantly between the two groups. Children with appendicitis were, however, admitted more often during daytime hours than children with enteritis. This may reflect that persistent abdominal pain prompts earlier presentation, whereas children with enteritis are often brought in during the evening or at night, after home management of vomiting and diarrhea has failed. This difference was not anticipated and may partly reflect referral patterns. Reports on the seasonality of pediatric appendicitis are mixed, and several series describe a summer peak that has been linked to the seasonal circulation of enteric viruses and to lymphoid hyperplasia of the appendix [20]. The absence of a clear seasonal signal in our data may reflect the modest sample size and the tertiary referral setting, and it should be interpreted with caution.

Management in our cohort was predominantly open appendectomy, which mirrors the tendency to operate on small children with advanced disease through an open approach, although data show that laparoscopic appendectomy is safe and is associated with favorable postoperative outcomes in children and is increasingly preferred [21]. The consistent use of the same surgical pathway, the analysis of all blood counts on a single hematology analyzer in the hospital laboratory, and the confirmation of every appendicitis diagnosis by histopathology strengthen the internal validity of these outcome data.

On the enteritis side, a causative pathogen was identified in 49 of 66 children, and the pattern was dominated by viruses, with rotavirus the leading agent, in keeping with its role as the most common cause of severe gastroenteritis in children younger than five years [22]. The relative lymphocytosis, the higher eosinophil counts, the higher platelet count, and the higher red cell distribution width seen in the enteritis group are consistent with a viral rather than a pyogenic inflammatory response and with the reactive thrombocytosis that frequently accompanies viral infection in early childhood [5,22]. Because the eosinophil values were higher only in comparison with the appendicitis group and were not related to age-specific reference ranges, they should not be interpreted as eosinophilia. Taken together with the biomarker profile, these microbiological findings support the biological plausibility of the separation we observed.

Our findings are consistent with a large body of evidence on the neutrophil-to-lymphocyte ratio in pediatric appendicitis. A systematic review and meta-analysis of nineteen studies and almost six thousand children reported a pooled sensitivity of 0.82 and a specificity of 0.76 with a summary area under the curve of 0.86 [12], values that are close to those we observed. The composite indices that combine the neutrophil, lymphocyte, platelet, and monocyte counts, namely the systemic immune-inflammation index and the systemic inflammatory response index, have more recently been shown to support the diagnosis of appendicitis in children [13,14,15], and in our data these indices also discriminated well, with areas under the curve of 0.85 and 0.87. Their practical value is that they capture the shift toward a neutrophilic and away from a lymphocytic profile in a single number derived from a test that is already performed at admission.

C-reactive protein showed the widest absolute separation between the two groups and, at 87.9%, the highest specificity among the individual markers. This is biologically expected because appendicitis is a bacterial and frequently transmural inflammatory process that produces a strong acute phase response, whereas most enteritis in this age group is viral and provokes little or no rise in C-reactive protein [5]. Previous pediatric work has nonetheless shown that a normal C-reactive protein alone cannot exclude early appendicitis, so its main value lies in confirming rather than excluding the diagnosis and in complementing the differential count [23]. Other acute phase reactants, such as fibrinogen, have also been evaluated for this purpose, with similar limitations [24].

An important consideration in the interpretation of these results is the similar age of the two groups. This contrasts with the usual epidemiology of the two diseases, since appendicitis is rare in infancy while gastroenteritis is most common in the first years of life [2,5]. The leukocyte differential changes physiologically with age, with a lymphocyte predominance in infancy that gradually shifts toward a neutrophil predominance during the preschool years. Because the two groups were of similar age, this normal age-related variation is unlikely to explain the observed difference in the neutrophil and lymphocyte fractions between the groups, which more likely reflects the disease process itself. C-reactive protein and the absolute magnitude of the inflammatory response are far less age-dependent, which strengthens their role as disease-specific markers, and the consistency of the neutrophilic signal across several independent indices argues that a genuine inflammatory difference is present.

The combination of C-reactive protein and the neutrophil-to-lymphocyte ratio outperformed any single marker, reaching an apparent area under the curve of 0.96 with a sensitivity of 96.3% and a negative predictive value of 95.0%. Predictive values, however, depend on the prevalence of appendicitis, which in our sample was determined by the selection of the two groups according to the final diagnosis. A high apparent negative predictive value therefore does not by itself constitute a safe strategy for ruling out appendicitis. In our data the two biomarkers also added discrimination beyond a simple clinical model based on abdominal pain, right lower quadrant tenderness, and diarrhea, although this comparison was exploratory and requires confirmation in an unselected cohort of children with diagnostic uncertainty. Similar multivariable approaches that combine clinical signs with the white cell count and C-reactive protein have been shown to distinguish appendicitis from gastroenteritis better than clinical scores alone [6].

A striking feature of our appendicitis group was that complicated appendicitis was present in almost four out of five children. This high proportion is consistent with the literature on preschool appendicitis, in which perforation rates rise steeply as age falls and exceed half of all cases in children younger than five years [17,19,25]. In our series, however, the proportion of complicated disease reflects the full histopathological spectrum, including gangrenous appendicitis without perforation, and should not be equated with the perforation rate, which was 38 of 80 children (47.5%). The late and nonspecific presentation, together with the thin appendiceal wall and the underdeveloped omentum of small children, favors early perforation, and delay in reaching the diagnosis rather than in-hospital delay appears to be the main driver [26]. The long hospital stay, the frequent need for drainage, and admission to intensive care in half of the children with appendicitis all reflect advanced disease already present at admission.

When we examined whether admission biomarkers could identify complicated appendicitis, most markers, including C-reactive protein and the neutrophil-to-lymphocyte ratio, did not distinguish complicated from uncomplicated disease. The immature granulocyte fraction and absolute count were the only markers that were higher in complicated appendicitis. This fits a growing body of work showing that immature granulocytes and the closely related delta neutrophil index rise with the severity of appendiceal inflammation and can help flag complicated or perforated disease in children [27]. The limited ability of the remaining markers to grade severity in our cohort probably reflects the fact that most children already had advanced inflammation at admission, which narrows the difference between the two subgroups. Because these differences did not remain significant after correction for multiple testing, discrimination was only moderate, and the comparison included only 17 children with uncomplicated disease, this finding should be regarded as exploratory.

Placed in the wider diagnostic context, our results complement meta-analytic evidence that the white cell count and C-reactive protein have only moderate accuracy for appendicitis when used alone [28], with population data showing that the burden of complicated disease is greatest in the youngest children [29], and with the recognition that imaging in young children is operator-dependent and often inconclusive [30]. A simple admission index that combines C-reactive protein with the neutrophil-to-lymphocyte ratio could therefore become an early and inexpensive aid in deciding which young children need prompt surgical review, but its effect on clinical decisions and patient outcomes has not been tested and requires prospective evaluation.

This study has several strengths. The diagnosis of appendicitis was confirmed intraoperatively and histopathologically, which provides a robust reference standard, the observation period spanned ten years, a comprehensive panel of primary and derived biomarkers was examined, correction for multiple testing was applied, the comparison group consisted of symptomatic children with enteritis, which is the clinically relevant comparison for this diagnostic question, and the laboratory platforms were the same as those used in our earlier pediatric work at this institution [31]. Several limitations should also be acknowledged. The design was retrospective and single-center. The two groups were identified independently by their final diagnosis rather than from a single cohort of children with undifferentiated acute abdominal pain, and the spectrum of differential diagnoses was limited to enteritis, which may overestimate diagnostic accuracy. Most children with appendicitis had complicated disease and most enteritis was viral, so the applicability of our findings to early appendicitis, to bacterial enteritis, and to children with an undifferentiated acute abdomen remains uncertain. Histopathology reports were issued by different pathologists over the study period without central review, and imaging, hydration status, and culture results were not analyzed systematically. C-reactive protein was measured on more than one analyzer during the study period, and the comparability of these assays was not formally assessed. The sample size did not allow a reliable comparison between infants younger than two years and older preschool children, a question that deserves a dedicated multicenter study. Finally, the cutoffs and the combined model were derived and optimized within this sample and were validated only internally, so they require external validation before they can be applied in routine practice.

Taken together, these findings suggest that inexpensive and universally available admission blood tests, in particular C-reactive protein and the neutrophil-to-lymphocyte ratio, may assist the difficult early distinction between acute appendicitis and acute enteritis in children younger than five years. Rather than replacing clinical judgment or imaging, these markers can be integrated with the history, the physical examination, and ultrasound to prioritize young children for surgical review or for observation, and might help reduce both delayed operations and unnecessary interventions [7,10], although such benefits were not assessed in our study and require prospective validation in consecutive children with diagnostic uncertainty. The same admission inflammatory profile may also help distinguish appendicitis from other common mimics of the acute abdomen in this age group, such as mesenteric lymphadenitis [32].

## 5. Conclusions

In children younger than five years, admission blood biomarkers are strongly associated with the final diagnosis of acute appendicitis or acute enteritis. A neutrophil-dominant profile, with an elevated neutrophil percentage, a high neutrophil-to-lymphocyte ratio, raised composite inflammatory indices, and markedly elevated C-reactive protein, points toward appendicitis, whereas relative lymphocytosis, higher eosinophil counts, and higher platelet counts favor enteritis. C-reactive protein and the neutrophil-to-lymphocyte ratio were the most informative individual markers, and their combination discriminated the two conditions with an apparent area under the curve of 0.96 that remained high after internal validation. Whether these simple and inexpensive tests, available at the moment of admission, improve clinical decision-making or patient outcomes in this difficult age group remains to be shown, and the proposed model and cutoffs require prospective multicenter validation in consecutive children with diagnostic uncertainty before clinical use.

## Figures and Tables

**Table 1:** Baseline demographic and clinical characteristics of the study population.

Characteristic	Acute Appendicitis (*n* = 80)	Acute Enteritis (*n* = 66)	*p*-Value
Age, months, median (IQR)	49 (40–54)	47 (38–53)	0.62
Male sex, *n* (%)	51 (63.8)	37 (56.1)	0.44
Duration of symptoms, hours, median (IQR)	42 (15–72)	24 (12–72)	0.38
Body temperature, °C, median (IQR)	37.8 (37–38.5)	37.2 (36.6–38.7)	0.10
Heart rate, per minute, median (IQR)	106 (94–121)	108 (96–123)	0.58
Respiratory rate, per minute, median (IQR)	23 (18–25)	24 (19–27)	0.41

**Table 2:** Clinical presentation of the study population.

Characteristic	Acute Appendicitis (*n* = 80)	Acute Enteritis (*n* = 66)	*p*-Value
Abdominal pain, *n* (%)	77 (96.3)	9 (13.6)	<0.001
Right lower quadrant tenderness, *n* (%)	44 (55.0)	20 (30.3)	0.004
Rebound tenderness, *n* (%)	27 (33.8)	0 (0.0)	<0.001
Nausea or vomiting, *n* (%)	52 (65.0)	40 (60.6)	0.61
Diarrhea, *n* (%)	16 (20.0)	35 (53.0)	<0.001
Bloody stools, *n* (%)	0 (0.0)	16 (24.2)	<0.001
Poor appetite, *n* (%)	40 (50.0)	34 (51.5)	0.87
Decreased urination, *n* (%)	8 (10.0)	4 (6.1)	0.55
Vomiting episodes, median (IQR)	3 (2–4.5)	4.5 (2–8)	0.11

**Table 3:** Seasonal and temporal distribution of admissions. Daytime was defined as the interval between 8 a.m. and 8 p.m. The *p*-value for season reflects the overall four-season distribution.

Characteristic	Acute Appendicitis (*n* = 80)	Acute Enteritis (*n* = 66)	*p*-Value
Winter, *n* (%)	20 (25.0)	16 (24.2)	0.22
Spring, *n* (%)	19 (23.8)	25 (37.9)	
Summer, *n* (%)	22 (27.5)	11 (16.7)	
Autumn, *n* (%)	19 (23.8)	14 (21.2)	
Daytime admission, *n* (%)	50 (62.5)	26 (39.4)	0.008

**Table 4:** Comparison of admission blood biomarkers between children with acute appendicitis and acute enteritis.

Biomarker	Acute Appendicitis, Median (IQR)	Acute Enteritis, Median (IQR)	*p*-Value	q-Value
Erythrocytes, ×10^12^/L	4.60 (4.33–4.79)	4.30 (3.93–4.78)	0.011	0.013
Hemoglobin, g/L	120.5 (113–125.3)	119 (111–128)	0.915	0.915
Hematocrit, L/L	0.37 (0.35–0.38)	0.36 (0.34–0.39)	0.843	0.873
MCV, fL	80.8 (78.4–82.3)	83.7 (78.4–92.2)	0.004	0.005
MCH, pg	26.5 (25.4–27.2)	27.9 (25.7–30.9)	0.002	0.003
MCHC, g/L	327 (321–331)	329 (321–338)	0.256	0.297
RDW-CV, %	13.2 (12.8–13.7)	14.0 (13.2–15.1)	<0.001	<0.001
Platelets, ×10^9^/L	297 (273–328)	399 (298–506)	<0.001	<0.001
MPV, fL	9.20 (8.88–9.43)	9.20 (8.80–9.78)	0.697	0.748
Leukocytes, ×10^9^/L	15.5 (12.2–18.9)	9.74 (6.98–15.09)	<0.001	<0.001
Neutrophils, %	78.4 (73.9–82.6)	50.2 (36.2–61.2)	<0.001	<0.001
Lymphocytes, %	13.1 (9.8–15.3)	37.0 (25.3–48.0)	<0.001	<0.001
Monocytes, %	7.45 (6.48–8.93)	10.8 (8.2–12.2)	<0.001	<0.001
Eosinophils, %	0.10 (0.00–0.30)	0.95 (0.20–2.30)	<0.001	<0.001
Basophils, %	0.20 (0.20–0.30)	0.30 (0.20–0.40)	<0.001	0.001
Immature granulocytes, %	0.40 (0.30–0.50)	0.20 (0.10–0.30)	<0.001	<0.001
Neutrophils, ×10^9^/L (absolute)	11.9 (9.4–14.9)	4.51 (2.90–8.07)	<0.001	<0.001
Lymphocytes, ×10^9^/L (absolute)	1.86 (1.39–2.66)	3.43 (2.14–5.92)	<0.001	<0.001
Monocytes, ×10^9^/L (absolute)	1.16 (0.85–1.43)	1.05 (0.76–1.39)	0.420	0.469
Eosinophils, ×10^9^/L (absolute)	0.02 (0.00–0.04)	0.10 (0.03–0.23)	<0.001	<0.001
Basophils, ×10^9^/L (absolute)	0.03 (0.02–0.04)	0.03 (0.02–0.04)	0.106	0.128
Immature granulocytes, ×10^9^/L (absolute)	0.06 (0.04–0.08)	0.02 (0.01–0.04)	<0.001	<0.001
Neutrophil-to-lymphocyte ratio	6.00 (4.85–8.28)	1.34 (0.77–2.41)	<0.001	<0.001
Platelet-to-lymphocyte ratio	156 (114–222)	99 (75–167)	<0.001	<0.001
Monocyte-to-lymphocyte ratio	0.60 (0.47–0.81)	0.31 (0.21–0.47)	<0.001	<0.001
Neutrophil-to-monocyte ratio	10.5 (8.0–12.0)	4.44 (2.68–6.10)	<0.001	<0.001
Systemic immune-inflammation index	1899 (1337–2611)	486 (297–864)	<0.001	<0.001
Systemic inflammatory response index	6.81 (4.28–11.79)	1.41 (0.70–3.06)	<0.001	<0.001
C-reactive protein, mg/L	81.2 (56.6–111.9)	4.0 (0.5–13.5)	<0.001	<0.001

**Table 5:** Diagnostic performance of the admission biomarkers with an area under the curve of at least 0.70 for differentiating acute appendicitis from acute enteritis, ranked by the area under the curve.

Biomarker	Higher in	AUC	Cutoff	Sensitivity, %	Specificity, %	PPV, %	NPV, %
C-reactive protein	Appendicitis	0.908	≥25.3	86.3	87.9	89.6	84.1
Neutrophils (%)	Appendicitis	0.904	≥65.0	88.8	81.8	85.5	85.7
Neutrophil-to-lymphocyte ratio	Appendicitis	0.898	≥3.12	87.5	83.3	86.4	84.6
Lymphocytes (%)	Enteritis	0.894	≤21.2	87.5	83.3	86.4	84.6
Neutrophil-to-monocyte ratio	Appendicitis	0.872	≥6.52	88.8	78.8	83.5	85.2
Systemic inflammatory response index	Appendicitis	0.868	≥3.60	81.3	83.3	85.5	78.6
Systemic immune-inflammation index	Appendicitis	0.852	≥888	87.5	77.3	82.4	83.6
Neutrophils (absolute)	Appendicitis	0.831	≥6.70	91.3	71.2	79.3	87.0
Immature granulocytes (absolute)	Appendicitis	0.799	≥0.05	68.8	81.8	82.1	68.4
Eosinophils (%)	Enteritis	0.788	≤0.30	78.8	69.7	75.9	73.0
Monocyte-to-lymphocyte ratio	Appendicitis	0.787	≥0.54	67.5	84.8	84.4	68.3
Monocytes (%)	Enteritis	0.754	≤10.1	87.5	60.6	72.9	80.0
Immature granulocytes (%)	Appendicitis	0.752	≥0.30	82.5	68.2	75.9	76.3
Lymphocytes (absolute)	Enteritis	0.752	≤3.14	86.3	54.5	69.7	76.6
Eosinophils (absolute)	Enteritis	0.750	≤0.08	86.3	54.5	69.7	76.6
Platelets	Enteritis	0.742	≤338	81.3	66.7	74.7	74.6
Leukocytes	Appendicitis	0.718	≥11.6	81.3	60.6	71.4	72.7

**Table 6:** Multivariable logistic regression model for the prediction of acute appendicitis.

Predictor	Adjusted Odds Ratio per 1 SD	95% Confidence Interval	*p*-Value
C-reactive protein	7.77	3.25–18.55	<0.001
Neutrophil-to-lymphocyte ratio	8.55	3.43–21.31	<0.001

Odds ratios are expressed per one standard deviation increase in the natural log-transformed value of each predictor. Adding the platelet count as a third predictor did not materially improve the model. SD, standard deviation. The model intercept was −4.7608, and the regression coefficients were 0.9000 for the natural log of C-reactive protein (mg/L) and 1.9451 for the natural log of the neutrophil-to-lymphocyte ratio.

**Table 7:** Admission blood biomarkers in complicated versus uncomplicated appendicitis.

Biomarker	Complicated (*n* = 63)	Uncomplicated (*n* = 17)	*p*-Value
Leukocytes, ×10^9^/L	15.5 (13.2–19.1)	14.1 (11.6–18.2)	0.51
Neutrophils, %	79.2 (72.9–84.1)	78.1 (76.1–79.1)	0.49
Neutrophil-to-lymphocyte ratio	6.29 (4.57–9.72)	5.52 (5.09–6.29)	0.29
Systemic immune-inflammation index	1923 (1192–2775)	1715 (1469–2057)	0.41
Systemic inflammatory response index	7.32 (4.51–12.55)	5.80 (4.17–8.49)	0.34
Immature granulocytes, %	0.4 (0.3–0.5)	0.3 (0.2–0.4)	0.008
Immature granulocytes, ×10^9^/L	0.06 (0.05–0.08)	0.04 (0.02–0.06)	0.026
C-reactive protein, mg/L	83.0 (64.5–127.1)	73.0 (25.7–104.6)	0.25
Platelets, ×10^9^/L	297 (271–332)	297 (281–327)	0.71
Length of hospital stay, days	9 (7.5–12)	6 (4.5–7.75)	0.004

## Data Availability

The data presented in this study are available on request from the corresponding author. The data are not publicly available due to privacy and ethical restrictions.

## Abbreviations

The following abbreviations are used in this manuscript:

AUC	Area under the curve
CBC	Complete blood count
CRP	C-reactive protein
ICD-10	International Classification of Diseases, tenth revision
IG	Immature granulocytes
IQR	Interquartile range
MCH	Mean corpuscular hemoglobin
MCHC	Mean corpuscular hemoglobin concentration
MCV	Mean corpuscular volume
MLR	Monocyte-to-lymphocyte ratio
NLR	Neutrophil-to-lymphocyte ratio
NMR	Neutrophil-to-monocyte ratio
NPV	Negative predictive value
PLR	Platelet-to-lymphocyte ratio
PPV	Positive predictive value
RDW-CV	Red cell distribution width, coefficient of variation
ROC	Receiver operating characteristic
SD	Standard deviation
SII	Systemic immune-inflammation index
SIRI	Systemic inflammatory response index
STROBE	Strengthening the Reporting of Observational Studies in Epidemiology
